# Independent factors associated with advanced testicular germ cell tumors: the roles of smoking, lymphovascular invasion, and tumor size

**DOI:** 10.1186/s12885-026-15598-y

**Published:** 2026-01-19

**Authors:** Selahattin Çelik, Engin Eren Kavak, İsmail Dilli, Esra Zeynelgil, Hatice Ayyıldız Sevim, Samed Rahatlı, Tülay Eren

**Affiliations:** Department of Medical Oncology, Ankara Etlik City Training and Research Hospital, Ankara, 06010 Turkey

**Keywords:** Testicular neoplasms, Germ cell and embryonal neoplasms, Lymphatic vessel invasion, Neoplasm size, Smoking, Obesity, Prognosis

## Abstract

**Background:**

Testicular germ cell tumors (TGCTs) are the most common solid malignancies among young men. Although overall survival exceeds 95%, a subset of patients still present with advanced disease or relapse. Identifying clinicopathological and modifiable lifestyle factors associated with advanced-stage presentation may improve risk stratification and guide management.

**Methods:**

This retrospective study included 96 patients with TGCTs treated at a single tertiary center. Data on smoking status, body mass index (BMI), lymphovascular invasion (LVI), and tumor size were analyzed. Associations between these parameters and stage at diagnosis were assessed using chi-square tests and multivariable logistic regression. Recurrence-free survival (RFS) was evaluated by the Kaplan–Meier method.

**Results:**

Among 96 patients (median age, 31 years), 59.4% had early-stage and 40.6% had advanced-stage disease. Smoking (OR = 8.17; *p* = 0.014), LVI (OR = 70.23; *p* < 0.001), and tumor size ≥ 4 cm (OR = 12.00; *p* = 0.009) were independently associated with advanced-stage presentation, whereas BMI showed no significant association. Recurrence occurred in 13.5% of patients, more frequently among smokers and LVI-positive cases, though not statistically significant.

**Conclusion:**

Smoking, lymphovascular invasion, and tumor size ≥ 4 cm were independently associated with advanced-stage presentation in TGCTs, whereas obesity showed no significant association. The combined evaluation of pathological and lifestyle factors may enhance individualized risk stratification and inform future risk-adapted management strategies.

## Introduction

Testicular germ cell tumors (TGCTs) are the most common solid malignancy in young adult men and have shown a steady increase in incidence worldwide over recent decades [1]. According to GLOBOCAN 2020 data, TGCTs account for approximately 1% of all male malignancies but remain the leading cancer type in men aged 15–44 years [2]. Because of early diagnosis, high chemosensitivity, and multidisciplinary management, overall survival now exceeds 95% [3]. Nevertheless, a subset of patients still present with advanced-stage disease or experience early recurrence, underscoring the need for refined prognostic markers.

The clinical staging, risk stratification, and treatment algorithms of TGCTs are well defined in the current ESMO-EURACAN and NCCN guidelines. These emphasize the integration of pathological and biological prognostic factors into clinical decision-making and advocate individualized post-orchidectomy management. For early-stage disease, active surveillance, single-agent carboplatin, or adjuvant radiotherapy may be considered according to risk profile, whereas advanced-stage cases are treated with platinum-based combination chemotherapy guided by the IGCCCG 2021 classification [4–6]. Despite these well-established systems, considerable clinical heterogeneity persists, underscoring the need for additional clinicopathologic and lifestyle-related predictors.

Among pathological parameters, lymphovascular invasion (LVI) is a well-established independent predictor of relapse in clinical stage I non-seminomatous TGCTs [7]. LVI, defined as the presence of tumor cells within lymphatic or vascular channels, marks an early step in metastatic dissemination. Multiple prospective and retrospective studies have demonstrated that the presence of LVI is associated with a significantly higher risk of relapse, with recurrence rates reaching up to 40–50% in LVI-positive patients compared with less than 15% in those without invasion [8, 9]. Consequently, LVI is a critical determinant in decisions regarding adjuvant chemotherapy and surveillance intensity. Current NCCN and ESMO guidelines recommend either a single cycle of BEP chemotherapy or close radiologic follow-up for patients with stage I non-seminoma and LVI positivity [4, 5]. Beyond its prognostic relevance, LVI reflects tumor aggressiveness and biological potential for metastasis.

Similarly, tumor size ≥ 4 cm has been established as an important morphological indicator associated with higher risk of metastatic spread and recurrence, particularly in seminomatous disease [10]. This threshold serves as a key criterion in both pathological and clinical staging systems. Larger tumors often correlate with increased tumor burden, necrosis, and a greater likelihood of lymphovascular invasion. In clinical stage I seminoma, this cut-off guides adjuvant carboplatin or radiotherapy recommendations, as lesions exceeding 4 cm carry a higher risk of occult metastasis. The concurrent evaluation of LVI and tumor size therefore provides valuable prognostic information supporting personalized treatment planning while avoiding overtreatment in low-risk patients [11].

The impact of modifiable lifestyle factors, particularly smoking and obesity, on TGCT development, stage at diagnosis, and outcomes has gained interest but remains incompletely defined. Cigarette smoking has been proposed as an environmental risk factor influencing both carcinogenesis and disease aggressiveness. Epidemiologic and clinical studies have reported an elevated TGCT risk and poorer survival among smokers [12, 13]. Mechanistically, tobacco-derived polycyclic aromatic hydrocarbons and nitrosamines induce oxidative DNA damage, p53 mutations, and impairment of DNA repair pathways, accompanied by increased pro-inflammatory cytokines and suppression of anti-tumor immune responses [14]. Nicotine exposure may also up-regulate hypoxia-inducible factor-1α (HIF-1α), promoting angiogenesis and tumor invasion [15].

In contrast, the association between obesity and TGCT is more ambiguous. Large-scale meta-analyses evaluating body-mass index (BMI) have revealed weak or inconsistent correlations with TGCT incidence. Obesity is thought to exert indirect effects through alterations in the hormonal and metabolic microenvironment, rather than through direct carcinogenicity. Adiposity-related estrogen-androgen imbalance, hyperinsulinemia, elevated insulin-like growth factor-1 (IGF-1), and chronic low-grade inflammation have been implicated as potential biological pathways facilitating germ-cell transformation. However, current evidence does not support obesity as an independent determinant of disease stage or recurrence risk [16, 17]. Thus, clarifying the prognostic relevance of adiposity in TGCTs remains an area of ongoing investigation.

Given these considerations, this study was designed as an exploratory, descriptive analysis to evaluate the association between modifiable lifestyle factors (smoking and obesity) and established pathological parameters (lymphovascular invasion and tumor size) with stage at diagnosis and recurrence in patients with testicular germ cell tumors. The primary objective was to characterize factors associated with advanced-stage presentation, while secondary objectives included exploratory assessment of recurrence patterns. This analysis was not intended to provide causal or practice-changing evidence, but rather to generate hypotheses for future prospective and multicenter studies.

## Materials and methods

### Study design and data collection

This retrospective study included patients diagnosed with TGCTs who were treated and followed at our institution. Eligible patients were adults diagnosed with histologically confirmed testicular germ cell tumors who underwent initial management and follow-up at our institution between 1 October 2022 and 1 September 2025. Patients with incomplete pathological data or missing staging information were excluded. Histologic subtypes were categorized as pure seminoma or non-seminoma. Clinical staging was based on the TNM classification and grouped as early stage (stage I) or advanced stage (stage II–III). Risk stratification for advanced disease followed the International Germ Cell Cancer Collaborative Group (IGCCCG) classification [4].

The following parameters were collected for each patient: age at diagnosis, date of diagnosis, smoking status (non-smoker or current/former smoker), laterality (right or left testis), body mass index (BMI, kg/m²), presence of comorbidities, tumor diameter (cm), histologic subtype, TNM stage, overall clinical stage, IGCCCG risk classification (good, intermediate, or poor risk), lymphovascular invasion (LVI; absent or present), first-line treatment modality (surveillance, single-agent carboplatin [AUC7], radiotherapy, BEP, EP, VIP, or RPLND), recurrence status, recurrence type and date, and last follow-up or death date.

Clinical stage was categorized as early stage (stage I, including subcategories 1a–1s) or advanced stage (stage II–III). BMI was evaluated both as a continuous variable and categorically as < 25, 25–29.9, or ≥ 30 kg/m². Tumor size was assessed using a 4 cm cutoff (< 4 cm vs. ≥4 cm). LVI was analyzed as a binary variable (absent vs. present).

This study was exploratory in nature and aimed to describe associations rather than establish causality or definitive prognostic models.

### Follow-up and outcome measures

Follow-up duration was calculated from the date of diagnosis to the date of last contact or death and expressed in months.

The primary endpoints were:

1. Stage at diagnosis (early vs. advanced), and.
2. Recurrence status (yes vs. no).

The secondary endpoint was recurrence-free survival (RFS), defined as the time from diagnosis to the date of recurrence. Patients without recurrence were censored at the last follow-up date.

### Statistical analysis

Continuous variables were summarized as mean ± standard deviation (SD) or median with interquartile range (IQR), and categorical variables as counts and percentages.

Group comparisons between early- and advanced-stage disease were performed using the chi-square test or Fisher’s exact test, as appropriate.

To determine independent predictors of advanced-stage presentation, a multivariable logistic regression model was constructed including smoking status, BMI (≥ 25 vs. <25 kg/m²), LVI (present vs. absent), and tumor size (≥ 4 cm vs. <4 cm) as covariates. Results were reported as odds ratios (ORs) with 95% confidence intervals (CIs) and p-values.

Recurrence-free survival (RFS) was estimated using the Kaplan–Meier method, and differences between groups were assessed by the log-rank test. Due to the limited number of recurrence events, only univariable analyses (chi-square and univariable logistic regression) were performed for recurrence-related comparisons.

All analyses were performed using IBM SPSS Statistics for Windows, version 26.0 (IBM Corp., Armonk, NY, USA). A p-value < 0.05 was considered statistically significant.

Given the exploratory nature of the study and the limited sample size, no formal sample size calculation or adjustment for multiple comparisons was performed, and the analyses should be interpreted as hypothesis-generating.

## Results

### Baseline characteristics

A total of 96 patients were included in the study, with a median age of 31 years (mean ± SD: 32.5 ± 9.7). Fifty-one patients (53.1%) had a BMI < 25 kg/m², 42 (43.8%) had a BMI between 25 and 29.9 kg/m², and 3 (3.1%) had a BMI ≥ 30 kg/m². Current smoking was reported in 53 (55.2%) patients, whereas 43 (44.8%) had never smoked.

The primary tumor involved the right testis in 51 (53.1%) and the left testis in 45 (46.9%) patients. Histologically, 40 (41.7%) cases were pure seminomas and 55 (57.3%) were non-seminomas. According to clinical stage, 57 (59.4%) patients presented with early-stage (stage I) disease and 39 (40.6%) with advanced disease (stage II–III). Based on IGCCCG risk classification, 85 (88.5%) patients were in the good-risk group, 2 (2.1%) in the intermediate-risk group, and 9 (9.4%) in the poor-risk group.

Tumor size was < 4 cm in 34 (35.4%) patients and ≥ 4 cm in 62 (64.6%). Lymphovascular invasion (LVI) was identified in 46 (47.9%) patients. Comorbidities were present in 31 (32.3%) and absent in 65 (67.7%) patients. The median follow-up duration was 18.1 months (IQR 15.6–23.0). During follow-up, 13 (13.5%) patients experienced recurrence, and one (1.0%) died.

Baseline demographic and clinical characteristics are summarized in Table 1.

**Table 1:** Baseline demographic and clinical characteristics (*n* = 96)

Age, years – mean ± SD (median)	32.5 ± 9.7 (31)
Smoking, n (%)	
Current Never	53 (55.2) 43 (44.8)
BMI, kg/m² – mean ± SD	25.1 ± 2.7
BMI, n (%)	
BMI < 25 BMI 25–29.9 BMI ≥ 30	51 (53.1) 42 (43.8) 3 (3.1)
Side, n (%)	
Right Left	51 (53.1) 45 (46.9)
Histology, n (%)	
Pure Seminoma Non-Seminoma	40 (41.7) 55 (57.3)
Stage, n (%)	
Early stage (I) Advanced stage (II–III)	57 (59.4) 39 (40.6)
Risk group, n (%)	
Good Intermediate Poor	85 (88.5) 2 (2.1) 9 (9.4)
Tumor size, n (%)	
< 4 cm ≥ 4 cm	34 (35.4) 62 (64.6)
LVI, n (%)	
No Yes	50 (52.1) 46 (47.9)
Comorbidity, n (%)	
Present Absent	31 (32.3) 65 (67.7)
Median follow-up, months (IQR)	18.1 (15.6–23.0)
Recurrence, n (%)	13 (13.5)
Death, n (%)	1 (1.0)

*BMI* Body Mass Index, *LVI* Lymphovascular invasion

### Association of modifiable and pathological factors with stage at diagnosis

In the early-stage group, 19 (33.3%) of 57 patients were smokers, compared with 34 (87.2%) of 39 patients in the advanced-stage group. The proportion of advanced disease was 11.6% among non-smokers and 64.2% among smokers (*p* < 0.001).

Patients with BMI ≥ 25 kg/m² had an advanced-stage rate of 35.6%, whereas those with BMI < 25 kg/m² had 45.1%; this difference was not statistically significant (*p* = 0.458).

Among patients without LVI, 96.0% were early stage and only 4.0% were advanced stage, whereas among those with LVI, 19.6% were early stage and 80.4% were advanced (*p* < 0.001).

Similarly, advanced disease was observed in 8.8% of patients with tumors < 4 cm compared with 58.1% in those with tumors ≥ 4 cm (*p* < 0.001).

The distribution of key clinicopathological variables by stage at diagnosis is presented in Table 2.

**Table 2:** Distribution of key risk factors by stage at diagnosis

Variable	Category	Early stage I (*n* = 57)	Advanced stage II–III (*n* = 39)	*p* value
Smoking	No	38 (88.4%)	5 (11.6%)	< 0.001
	Yes	19 (35.8%)	34 (64.2%)	
BMI	< 25	28 (54.9%)	23 (45.1%)	0.458
	≥ 25	29 (64.4%)	16 (35.6%)	
LVI	No	48 (96.0%)	2 (4.0%)	< 0.001
	Yes	9 (19.6%)	37 (80.4%)	
Tumor size	< 4 cm	31 (91.2%)	3 (8.8%)	< 0.001
	≥ 4 cm	26 (41.9%)	36 (58.1%)	

*Chi-square test

*BMI* Body Mass Index, *LVI* Lymphovascular invasion

### Multivariable analysis

In multivariable logistic regression analysis, smoking (OR = 8.17; 95% CI: 1.53–43.58; *p* = 0.014), presence of LVI (OR = 70.23; 95% CI: 10.78–457.48; *p* < 0.001), and tumor size ≥ 4 cm (OR = 12.00; 95% CI: 1.87–76.92; *p* = 0.009) were independently associated with an increased likelihood of presenting with advanced-stage disease.

BMI ≥ 25 kg/m² was not significantly associated with disease stage (OR = 1.17; 95% CI: 0.24–5.82; *p* = 0.847).

Detailed results of the multivariable model are shown in Table 3.

**Table 3:** Multivariable logistic regression analysis of factors associated with advanced stage (II–III) at diagnosis

Variable	OR	95% CI	*p* value
Smoking (yes)	8.17	1.53–43.58	0.014
Lymphovascular invasion (present)	70.23	10.78-457.48	< 0.001
Tumor size ≥ 4 cm	12.00	1.87–76.92	0.009
BMI ≥ 25 kg/m²	1.17	0.24–5.82	0.847

Reference categories: non-smoker, no lymphovascular invasion, tumor size < 4 cm, BMI < 25 kg/m²

### Recurrence and related factors

**Fig. 1:**
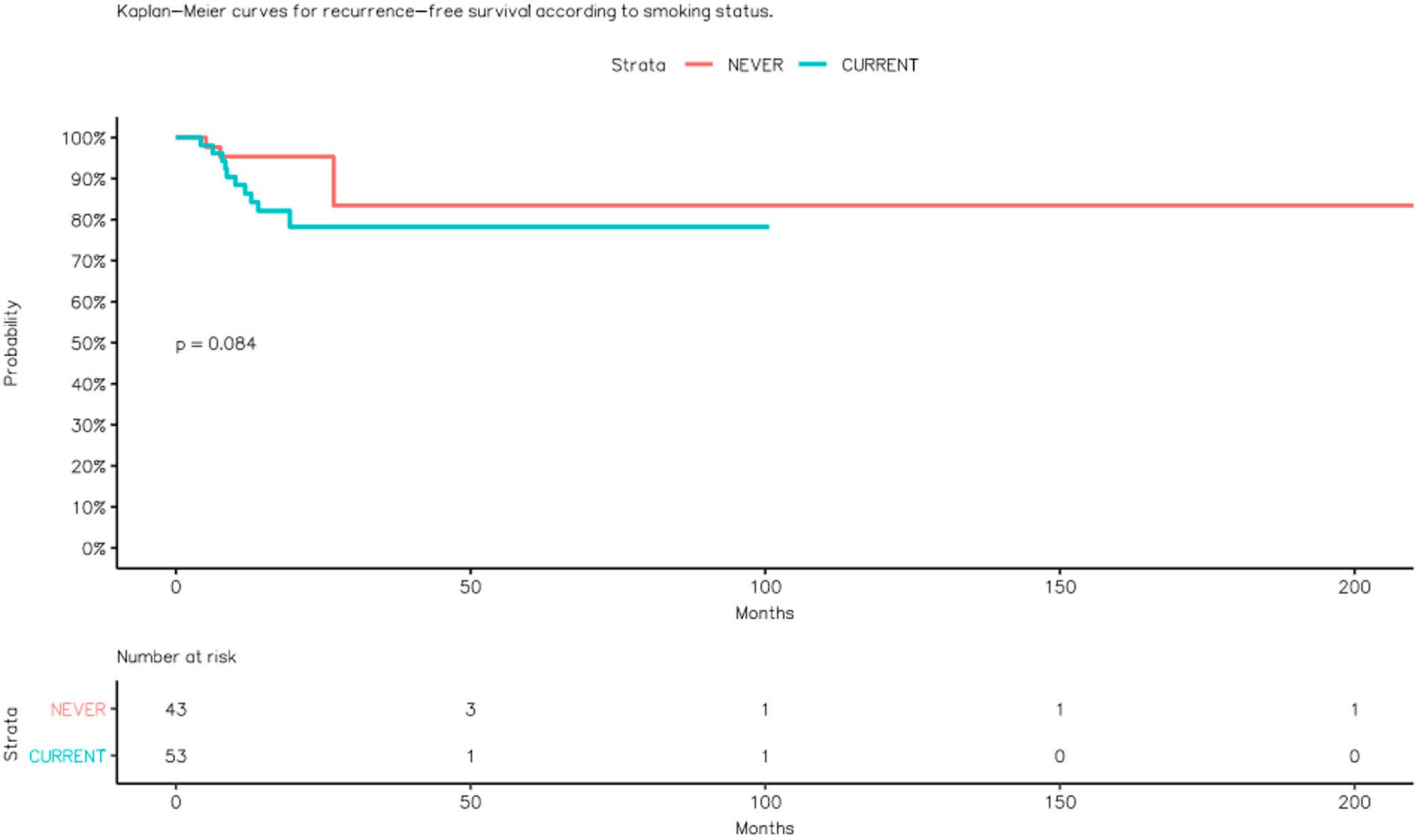
Kaplan–Meier curves for recurrence-free survival according to smoking status

During follow-up, 13 (13.5%) patients developed recurrence, with a median time to recurrence of 8.5 months. Recurrence occurred in 7.0% (3/43) of non-smokers and 18.9% (10/53) of smokers (Fig. 1).

**Fig. 2:**
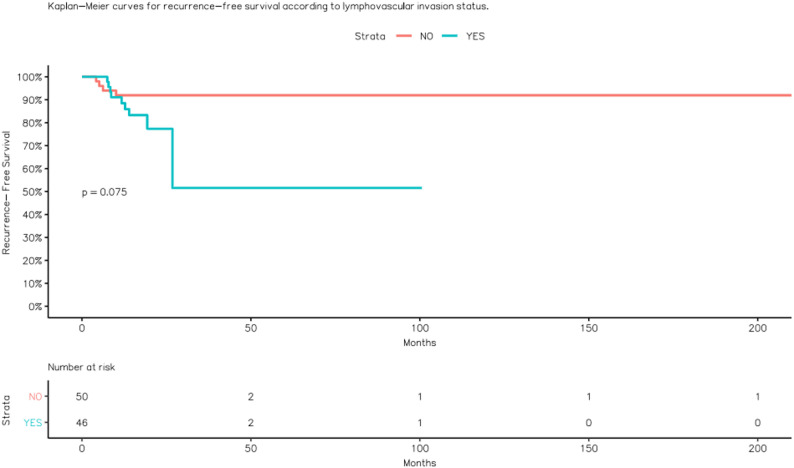
Kaplan–Meier curves for recurrence-free survival according to lymphovascular invasion status

Among patients without LVI, recurrence was observed in 8.0% (4/50), compared with 19.6% (9/46) among those with LVI (Fig. 2).

Recurrence occurred in 17.6% of patients with BMI < 25 kg/m² and 8.9% of those with BMI ≥ 25 kg/m². Regarding tumor size, recurrence rates were 14.7% for < 4 cm and 12.9% for ≥ 4 cm lesions. Although recurrence tended to be more frequent among smokers and LVI-positive cases (OR ≈ 3.10 for smoking; OR ≈ 2.80 for LVI), these associations did not reach statistical significance, likely due to the limited number of recurrence events.

Recurrence-related findings are summarized in Table 4.

**Table 4:** Recurrence rates according to modifiable and pathological factors

Variable	Category	No recurrence *n* (%)	Recurrence *n* (%)	Recurrence rate (%)
Smoking	No	40	3	7.0
	Yes	43	10	18.9
BMI	< 25	42	9	17.6
	≥ 25	41	4	8.9
LVI	Absent	46	4	8.0
	Present	37	9	19.6
Tumor size	< 4 cm	29	5	14.7
	≥ 4 cm	54	8	12.9

*BMI* Body Mass Index, *LVI* Lymphovascular invasion

## Discussion

In this study, we investigated the association between modifiable lifestyle factors and pathological parameters with stage at diagnosis and recurrence in patients with TGCTs. Smoking, lymphovascular invasion, and tumor size ≥ 4 cm were independently associated with advanced-stage presentation, whereas BMI showed no significant association.Although recurrence was more frequent among smokers and LVI-positive patients, this difference did not reach statistical significance, likely due to the limited number of events. This might reflect the short median follow-up of 18 months.

LVI has long been recognized as one of the most powerful predictors of relapse in stage I non-seminomatous TGCTs [7]. In our cohort, its presence was associated with an approximately 70-fold higher likelihood of presenting with advanced disease, emphasizing its role as a marker of tumor aggressiveness and early dissemination. Previous studies have consistently shown recurrence rates of up to 40–50% in LVI-positive patients compared with less than 15% in those without invasion [8, 9]. This finding supports the integration of LVI status into individualized post-orchidectomy management algorithms, as endorsed by ESMO and NCCN guidelines [4, 5]. LVI may also reflect tumor–stroma and endothelial interactions that facilitate metastatic spread [18].

Tumor size ≥ 4 cm was another strong pathological determinant of advanced disease. This threshold has been incorporated into both seminomatous and non-seminomatous risk classifications [10, 11]. Larger tumors correlate with higher tumor burden, necrosis, and a greater likelihood of micro-metastatic spread. Our findings are consistent with those of Boormans et al., who demonstrated that tumor diameter ≥ 4 cm combined with rete testis invasion predicted relapse in clinical stage I seminoma [11]. Similarly, Dieckmann et al. reported strong correlations between primary tumor size, histology, and tumor-marker elevation [10]. Together, these observations underscore the importance of tumor size as an accessible and reproducible indicator of biological aggressiveness.

Despite these limitations, the study has notable strengths, including a well-defined and uniformly treated patient population, standardized pathological evaluation, and the combined assessment of smoking status, lymphovascular invasion, and tumor size as factors associated with advanced-stage disease.

The relationship between smoking and advanced-stage disease was one of the most striking observations in our study. Smokers had more than eightfold higher odds of advanced-stage TGCT at diagnosis compared with non-smokers. This finding corroborates the report by Bandak et al., who identified smoking as an independent adverse prognostic factor for survival in disseminated disease [12]. Mechanistically, tobacco-derived carcinogens such as polycyclic aromatic hydrocarbons and nitrosamines induce oxidative DNA damage, p53 mutations, and impaired DNA-repair capacity [14], while nicotine up-regulates HIF-1α, promoting angiogenesis and invasion [15]. Chronic systemic inflammation and immune suppression associated with smoking may further facilitate tumor progression [19]. These mechanisms plausibly explain the association between smoking and a more aggressive clinical presentation.

Conversely, obesity was not significantly related to disease stage in our series. Although adiposity affects hormonal balance and insulin sensitivity, epidemiologic studies have produced inconsistent findings. Large meta-analyses have shown that BMI does not independently predict TGCT risk or outcomes [16, 17]. Our results are consistent with these reports, suggesting that obesity may exert indirect effects through metabolic or endocrine alterations rather than direct tumor-promoting mechanisms. Recent evidence further indicates that metabolic dysregulation and insulin resistance may affect long-term survivorship rather than primary tumor behavior [20].

Integrating modifiable lifestyle factors with established pathological predictors provides a more comprehensive view of TGCT biology. Smoking history, an easily obtainable clinical variable, could complement traditional prognostic tools such as the IGCCCG classification [6]. Incorporating such variables into clinical risk models may help refine treatment decisions and follow-up strategies [21]. Overall survival from TGCT has markedly improved over recent decades, largely due to risk-adapted therapy and multidisciplinary management [22]; however, identifying patients at risk for advanced or recurrent disease remains critical to optimizing long-term outcomes.

Several limitations of this study should be acknowledged. The retrospective, single-center design may limit the generalizability of the results. The limited sample size and number of events reduced statistical power and precluded extensive multivariable modeling, particularly for recurrence-related analyses. Additionally, no correction for multiple comparisons was applied, increasing the possibility of type I error.

Moreover, BMI may not accurately reflect adiposity-related risk, as it does not differentiate between lean and fat mass or account for fat distribution. Residual confounding by unmeasured clinical variables and lack of external validation further limit the generalizability of the findings.Future multicenter, prospective studies with larger patient cohorts and more precise metabolic assessments—such as visceral fat index or waist-to-hip ratio—are warranted to validate these findings. Despite these limitations, the study has notable strengths, including a well-defined and uniformly treated patient population, standardized pathological evaluation, and the novel combined assessment of smoking status, LVI, and tumor size was associated with advanced disease. This integrated evaluation enhances the clinical relevance and robustness of the results and provides a more comprehensive understanding of how pathological and lifestyle factors jointly influence tumor behavior.

In conclusion, Smoking, lymphovascular invasion, and tumor size ≥ 4 cm were associated with advanced-stage presentation in testicular germ cell tumors, whereas obesity showed no significant prognostic impact. While incremental, this real-world analysis provides hypothesis-generating insights that may inform future prospective or multicenter studies.

## Data Availability

The datasets generated and analyzed during the current study are available from the corresponding author upon reasonable request.
